# A Closer Look at Parental Anxiety in Asthma Outpacing Children’s Concerns: Fear of Physical Activity over the Fear of Drug Side Effects

**DOI:** 10.3390/children11030289

**Published:** 2024-02-29

**Authors:** Marijana Rogulj, Katarina Vukojević, Linda Lušić Kalcina

**Affiliations:** 1 Pediatric Clinic, University Hospital Center Split, 21000 Split, Croatia; 2 School of Medicine, University of Split, 21000 Split, Croatia; 3 Department of Anatomy, Histology and Embryology, School of Medicine, University of Split, 21000 Split, Croatia; katarina.vukojevic@mefst.hr; 4 Department of Neuroscience, School of Medicine, University of Split, 21000 Split, Croatia; llusic@mefst.hr; 5 Department of Psychology, Faculty of Humanities and Social Sciences in Split, University of Split, 21000 Split, Croatia

**Keywords:** asthma, anxiety, side effects, physical activity, asthma control

## Abstract

Background: The recognition of comorbidities is relevant for asthma management, especially if these conditions/diseases are treatable traits such as anxiety. This study aimed to explore the associations between asthma severity and child and parent asthma-related anxiety and to recognize the most common specific fears. Methods: This cross-sectional study consisted of 150 parents and their children diagnosed with asthma, and was conducted at the Pediatric Clinic of the University Hospital Center Split in Croatia. All children, from ages 3 to 17 years, underwent a thorough clinical examination. A total of 150 parents and 108 children filled out an asthma-related anxiety questionnaire in paper form. Results: Parents of children with moderate and severe asthma had higher asthma-related anxiety due to restrictions related to asthma symptoms (*p* = 0.032), and children diagnosed with moderate and severe asthma had greater anxiety due to restrictions related to asthma symptoms than children diagnosed with mild asthma (*p* = 0.004). Children’s anxiety was the highest when they experienced an asthmatic attack during physical activity (PA), and they fear that they will not be successful in sports or dancing due to asthma. Parents commonly reported the fear of an asthma attack without warning signs (*p* < 0.001), fear of drug side effects (*p* < 0.001), fear of absence from school (*p* = 0.006), and fear of an asthma attack during PA (*p* < 0.001). Conclusions: The current study reports findings of increased parental levels of anxiety when compared to their children, related to fear of an asthma attack occurring without warning signs, fear of side effects and fear of absence from school, as well as the fear of an asthma attack occurring during sports activities. When assessing individual items on anxiety associated with asthma, children most commonly reported concern related to physical activity.

## 1. Introduction

Asthma is the most common pediatric chronic illness, with significant levels of morbidity and mortality among children worldwide [1,2]. According to the recent literature, the asthma prevalence in the population is 6.16% [3]. In Croatian children younger than 14, the prevalence of asthma is 4.66% and it is 4.61% for all ages [3]. Available data provide an insight into the global burden of asthma [4,5], obliging everyone involved in the treatment of children with asthma to further improve care. The basis of asthma treatment is drug treatment, and this includes maintenance therapy and alleviation therapy. Maintenance therapy consists of basic drugs (controllers) that are taken over a longer period of time to achieve long-term control of inflammation, such as inhaled and systemic corticosteroids, leukotriene antagonist receptors, long-acting β2-agonists (LABAs) and monoclonal antibodies or biological therapy. Alleviation therapy consists of symptomatic drugs (relievers), such as short-acting β2-agonists (SABAs) and other bronchodilators [2].

Along with drug treatment, the recognition of associated comorbidities in asthma patients is considered very important for the treatment of asthma, especially if these conditions can be treated. The recognition of such treatable traits is especially important in severe asthma and represents a new strategy in individualized treatment programs [6]. Taking into account that the common predictors of the risk of exacerbation in severe asthma are extrapulmonary properties [6], recognizing such conditions is recommended in asthma patients. 

Anxiety in patients diagnosed with asthma is one of such treatable traits in asthma with a high prevalence [6,7]. Anxiety and depression are up to six times more common in people with asthma than in those without asthma, and asthma sufferers have an anxiety prevalence rate of 11 to 37%. Despite such a high prevalence, psychological comorbidities in asthma are still underdiagnosed and undertreated. Anxiety in asthma patients has also been associated with poor quality of life [8] and exacerbation of disease [9,10,11], and a number of pathophysiological mechanisms have been proposed associating anxiety with worse asthma outcomes. Additionally, the cornerstone in treatment is inhaled corticosteroids (ICSs) [2], a therapy that has been causing serious concerns and anxiety in parents. Such concerns have often been leading to treatment non-adherence [12], therefore interfering with maintenance therapy and alleviation therapy in pediatric asthma patients.

It has also been suggested that asthma patients often avoid exercise activity due to fears of worsening symptoms during physical effort [13,14], even though a reduced physical activity (PA) has been associated with increased disease severity, worse asthma control and an increased risk of exacerbation, significantly affecting the occurrence of obesity, which also affects asthma outcomes [15,16]. In addition to the aforementioned concerns, recognizing other specific triggers of anxiety in both children with asthma and their parents is mandatory in order to plan interventions and enable better asthma outcomes in children.

Therefore, the aim of this study is to assess the anxiety related to asthma in children with asthma and in their parents, as well as to describe the most commonly reported factors contributing to overall asthma-related anxiety in both groups. It is of particular interest to see the incidence of anxiety associated with the fear of drug side effects and the fear of children’s PA. 

## 2. Materials and Methods

### 2.1. Participants and Setting

This cross-sectional study was conducted between May 2021 and May 2022 at the Pediatric Clinic of the University Hospital Center Split in Split, Croatia, and included 150 children and youth with asthma (3 to 17 years old) and their parents. During prescheduled visits to a pediatric allergologist and after a regular clinical examination and examination of anamnestic data on asthma control and spirometric testing, the doctor presented the research.

Parents and children with a diagnosis of asthma were asked to participate, and the pediatrician consecutively informed parents (*n* = 153) about the study. A total of 98% (*n* = 150) of the parents answered the questionnaire. Exclusion criteria were children whose parents or legal guardians refused to sign informed consent, comorbidities with other chronic diseases under medical treatment and children who have been diagnosed with asthma for less than 6 months. There were no legal guardians of the children in the study. Following the clinical visit, parents completed a survey with demographic factors (i.e., age, gender) and filled out a printed paper form of the questionnaire. Children who could understand the questionnaire (over 9 years old) and who agreed to the examination filled out a printed paper form of the questionnaire independently in the presence of their parents (*n* = 108).

The study protocol was approved by the Institutional Review Board of the University Hospital Center Split in Split, Croatia (protocol #500-03/21-01-127), and the collection of the data from patients and their parents began after ethical approval.

### 2.2. Asthma Severity 

Based on the Global Initiative of Asthma (GINA) guidelines, the level of asthma symptom control for 4 weeks prior to the visit was assessed—symptoms of asthma during the day, symptoms of asthma during the night, the child’s level of activity and the need for reliever therapy of a short-acting beta agonist (SABA). During the clinical visit, the doctor asked the children and parents about the daytime and nighttime symptoms, about the need for relievers when symptomatic and about PA limitations. The required pharmacological regimen (types of drugs, doses and frequency of administration) was assessed. Spirometry was performed by a trained nurse using the Schiller SpiroScout LF8 in order to measure the following: vital capacity (VC); forced vital capacity (FVC); forced expiratory volume in 1 s (FEV1); forced expiratory flow at 50%FVC (FEF50); and peak expiratory flow (PEF) [17]. The interpretation of the spirometry results was performed by a pediatric allergologist. Based on a clinical examination, anamnestic data on asthma control and obtained spirometric results enabled the classification of the child’s asthma into one of five stages as follows: mild asthma (stages 1 and 2); moderate asthma (stages 3 and 4); and severe asthma (stage 5).

### 2.3. Anxiety 

Asthma-related anxiety was included in the study as a possible predictor and measured with a validated test. Children aged 9 years and older completed the Youth Asthma-Related Anxiety Scale (YAAS) and parents completed the Parent Asthma-Related Anxiety Scale (PAAS) [18]. The YAAS assesses how often the children worried about their asthma over the past two weeks. It includes 9 items, where children assess fear on a 6-point scale ranging from 0 (never) to 5 (always). The items include the assessment of fear regarding an asthma attack happening suddenly, the fear of an asthma attack happening while the child does not have medication, the fear of dying, the social anxiety (what would their friends think if the asthma attack happens), the fear of side effects, the fear of missing school and the fear of not keeping up with others, as well as the fear of not keeping up with others in sports and the fear of physical activity. The PAAS is a parallel parent version, including 11 items for the assessment of parents’ anxiety regarding their child’s asthma, using the same Likert scale assessment as the YAAS [18]. Items include similar aspects of anxiety as in the YAAS, assessed from the parental perspective, and include two items on the assessment of fear of an attack happening to the child while the parent is not with the child and the fear of the child not knowing the appropriate reaction to an asthma attack. Translation from English to Croatian and back to English was performed by 3 bilingual translators in each step, according to guidelines, and psychometric properties showed internal consistency of 0.80 for the YAAS and a Cronbach α of 0.86 for the PAAS scale in Croatian.

### 2.4. Statistical Analyses

Data analyses were performed using SPSS trial version (IBM Corp. 2020. IBM SPSS statistics for Windows, version 27.0. Armonk, NY, USA: IBM Corp) and JASP (JASP 2022 Version 0.16.2 computer software). The sample size required for the research was calculated based on the value of the correlation coefficient of the results obtained on a sample of 134 children diagnosed with asthma, and the correlation coefficient used to calculate the sample size represents the association between the scores of children diagnosed with asthma who participated in a previously published study by the authors of the questionnaire [18], using a scale of anxiety related to asthma, with the results presented on a scale of asthma severity. The value of the correlation coefficient was used to calculate the effect size; when the α value was set to 0.05, the statistical power calculated as 1-β was set to 0.95. The minimal sample size required for the planned research, according to the calculation based on the values set in the G*Power Statistical Power Analyses program for Mac and Windows, was 70 subjects. All the results are presented as frequencies (percentages) for categorical variables, and as means ± standard deviations for continuous variables. *t*-tests for independent samples with an appropriate assumption of homogeneity of variances were performed for the comparison of patients with mild asthma vs. patients with moderate to severe asthma, following the classification of patients in these two groups based on the GINA scale assessment. A *t*-test for paired samples was performed on a total of 108 respondents, where both the parent and the child filled out the questionnaire. Average values achieved on individual items of the PAAS and YAAS scales are presented in charts as means ± standard deviations for the full sample. The comparison of responses to specific questions compared in the PAAS and YAAS scales was assessed with the Mann–Whitney non-parametric test. Psychometric properties are reported as Cronbach α calculations for the PAAS and YAAS scales. Significant differences were considered for *p* < 0.05.

## 3. Results

A total of 72% of children were diagnosed with mild asthma, and 5.3% of patients had severe asthma. This study included 150 parents of children diagnosed with asthma and a total of 108 children diagnosed with asthma filled out the questionnaire on asthma-related anxiety. The median age of the children was 11 years old (8–13 IQR). The youngest child included in the study was 3, and the oldest was 17 years old, with 52% of them male.

Table 1 details the children’s demographic characteristics and clinical status. Asthma symptom control was assessed with the use of questions about daytime and nighttime symptoms in the last four weeks, limitation of PA and reliever (SABA) medication use, according to the GINA guidelines. The pharmacological regimen, including types of medicine, dosages and frequencies of administration, classified the child’s asthma as mild asthma (stages 1 and 2), moderate asthma (stages 3 and 4) or severe asthma (stage 5). Most of the children were treated with ICS as monotherapy (60.7%), both ICS and LABA therapy (14%) or with both ICS and montelukast therapy (8.7%). Biological therapy was prescribed to a total of three patients (2%). Therefore, 66.7% of patients were classified as GINA stage 2, and 22.7% of patients were classified as GINA stages 3 or 4. 

The full score on the PAAS scale in the total sample was 1.51 ± 0.83, whereas the full score on the YAAS scale reported by children was 1.02 ± 0.77 (Table 1).

Children diagnosed with moderate and severe asthma reported higher levels of overall anxiety associated with asthma compared to children diagnosed with mild asthma (1.24 ± 0.75 vs. 0.92 ± 0.77; *p* = 0.047). Significantly higher indicators of asthma-related anxiety were reported in parents of children with moderate and severe asthma on the anxiety subscale due to restrictions related to asthma symptoms (*p* = 0.032, Table 2). 

**Figure 1:**
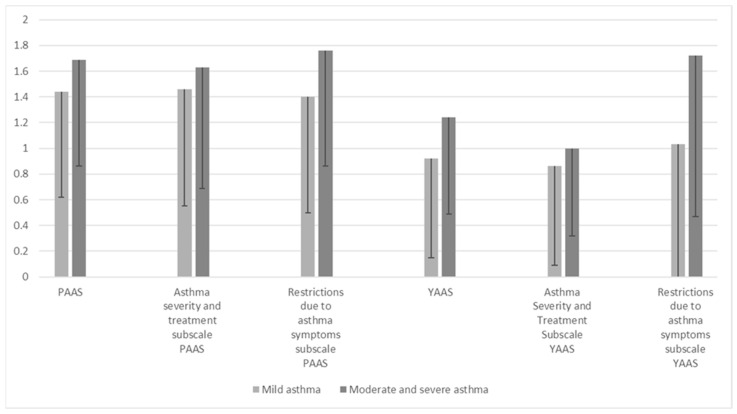
Comparison of total scores achieved on the YAAS (*n* = 108) and PAAS (*n* = 150) and subscales of children and parents of children diagnosed with mild versus moderate and severe asthma.

Similarly, children diagnosed with moderate and severe asthma had greater anxiety due to restrictions due to asthma symptoms (1.72 ± 1.25) than children diagnosed with mild asthma (1.03 ± 1.04, *p* = 0.004, Table 2, Figure 1).

**Figure 2:**
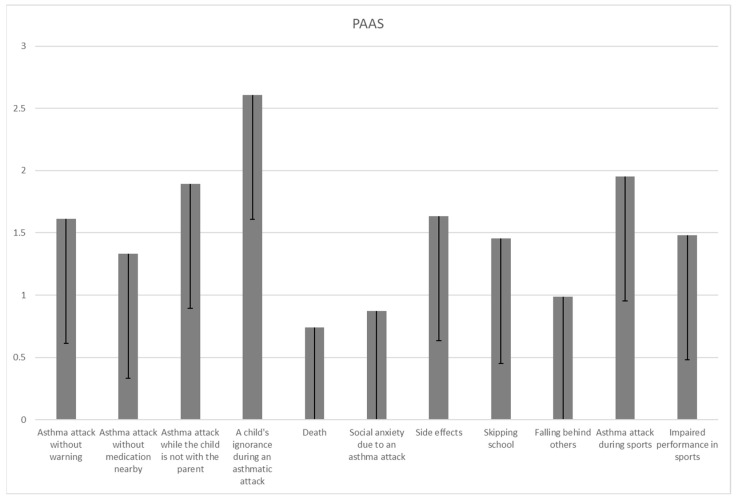
Average values achieved on individual items of the PAAS (*n* = 150).

**Figure 3:**
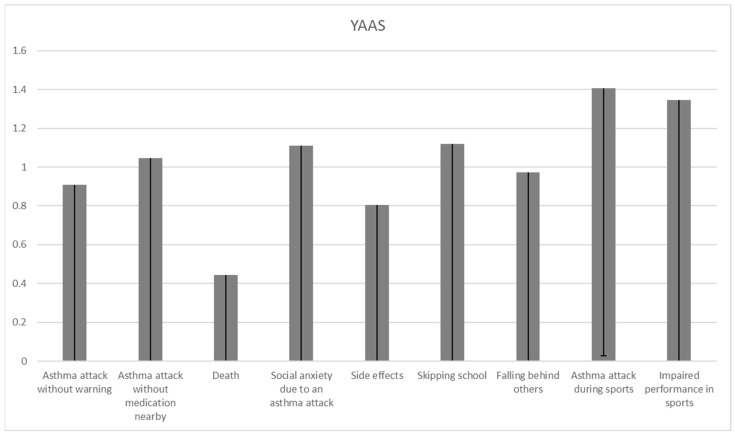
Average values achieved on individual items of the YAAS (*n* = 108).

The results of individual items of the YAAS and PAAS in Figure 2 and Figure 3 indicate the variability in parent and children anxiety. Parental anxiety was most frequently reported on items related to the fear that the child has an asthma attack while the parent is not with them (1.9 ± 1.3), that the child will not know what to do if they have an asthmatic attack while the parent is not with them (2.6 ± 1.7) and that the child will have an asthmatic attack during PA (2 ± 1.3). Parents often reported the fear that the child would have side effects from asthma medication (1.6 ± 1.3), and the parental fear of an asthma attack happening during PA was more commonly reported than the fear of side effects (*p* = 0.026).

The children’s anxiety on individual items of the YAAS reported in Figure 3 indicates common reports of fear that the child will have an asthmatic attack during PA (1.4 ± 1.4) and the fear that they will not be successful in sports or dancing due to asthma (1.3 ± 1.5). The child’s fear of an asthma attack happening during PA was more commonly reported than the fear of side effects (*p* = 0.047).

In order to compare the intensity of self-reported parental and children’s fear in specific items comparable in the YAAS and PAAS, differences in specific items are presented in Table 3. 

Parents reported higher levels of anxiety related to fear of an asthma attack occurring without warning signs (1.6 ± 1.3 vs. 0.9 ± 1; *p* < 0.001), fear of side effects (1.6 ± 1.3 vs. 0.8 ± 1; *p* < 0.001), fear of the child missing out in school (1.5 ± 1.3 vs. 1.1 ± 1.4; *p* = 0.006), and the fear of an asthma attack occurring during PA (2 ± 1.3 vs. 1.4 ± 1.4; *p* < 0.001). Children and parents reported a similar level of fear regarding situations when asthma attacks could happen while not having medication, the fear of death and the fear of social anxiety due to an asthma attack happening in public (what the friends of the child would think). No differences were also found in the overall level of anxiety between children and their parents in the fear of falling behind (not keeping up with others because of asthma) and the fear of impaired performance in sports and dancing (Table 3). There was no item suggesting a higher level of asthma-related fear in children compared to their parents.

## 4. Discussion

This study aimed to investigate the most commonly reported fears in both children with asthma as well as their parents, taking into account the severity of asthma in children. Parents reported higher levels of anxiety than children in items related to fear of an asthma attack occurring without warning signs, fear of side effects, fear of the child missing out in school, and fear of an asthma attack occurring during sports activities. When assessing individual items on anxiety associated with asthma, children most commonly reported concern related to PA and fear of not being successful in sports or dancing due to asthma, while parents most commonly reported fear that the child will not know what to do if they have an asthma attack while the parent is away. Taking asthma severity into account, the findings of this study suggest that children diagnosed with moderate and severe asthma had greater overall asthma-related anxiety as well as anxiety due to restrictions related to asthma symptoms when compared to children diagnosed with mild asthma. On the other hand, parents of children diagnosed with different asthma severities reported high levels of overall asthma-related anxiety regardless of asthma severity, and parents of children with mild and moderate asthma reported more anxiety than others only regarding restrictions related to asthma symptoms. 

The reported findings provide an insight into the anxiety of both children with asthma and their parents, previously recognized as a treatable trait and considered very important for asthma management. New findings on such treatable traits in asthma are a relevant step in planning individualized treatment programs [19,20]. The current findings are especially relevant since anxiety in asthma has been previously associated with poorer asthma control, a higher frequency of exacerbations, higher doses of drugs that asthma patients are treated with and poorer compliance to therapy [9,10,11].

Studies have confirmed an increased overall anxiety in parents of children diagnosed with asthma [21], and this has been associated with poor compliance with treatment with inhaled corticosteroids, as well as with an increased severity of asthma in children [22] and poorer asthma self-management [23]. The current study provides evidence of parental asthma-related anxiety regardless of asthma severity, and of higher levels of parental anxiety than children’s anxiety related to the fear of an asthma attack occurring without warning signs, fear of side effects, fear of the child missing out in school and fear of an asthma attack occurring during sports activities. Therefore, such fears should be addressed in further research in order to plan interventions and enable better asthma outcomes in children. The blunted response of the hypothalamic-pituitary-adrenal axis (HPA axis) in children with asthma may be caused by the decreased sensitivity of steroid receptors after chronic stress related to asthma [24,25,26,27], and since parental anxiety may affect the child’s level of stress, it is relevant to intervene in parental behaviors occurring as a consequence of asthma-related anxiety. 

One of the most important components of parents’ anxiety recognized in this study is the fear of the drugs their children are being treated with. Fear of side effects was more frequently reported in parents than in children with asthma. Inhaled corticosteroids (ICSs) have been the main approach in asthma therapeutics for more than half a century [28,29], in particular for those with the treatable trait of eosinophilic airway inflammation [30]. They have improved the quality of life and saved lives due to numerous treatment benefits as well as their safety profile [31]. This study suggests that parental fears regarding drug side effects should be addressed by clinicians and all experts working with pediatric asthma patients, and that parental anxiety regarding asthma severity and treatment is not dependent on the severity of the disease itself. 

The most commonly reported children’s concern recognized in the current study is the fear related to PA and the fear of not being successful in sports or dancing due to asthma. Such a common fear should be well addressed with interventions, since insufficient movement and a sedentary lifestyle further reduce physical fitness and actually lower the threshold for exercise-induced bronchoconstriction (EIB) [32]. PA in asthma is known to be associated with better asthma control, reduced markers of systemic inflammation and an improved lung function and thus exercise capacity [20,33,34], and it may significantly affect quality of life and mental health [35]. Parents in this study reported the fear of an asthma attack happening during sports even more commonly than children did. Considering the earlier research results, which suggest that parents use a reactive asthma management style and control their child’s asthma by avoiding potential triggers [36], interventions aimed at parents’ knowledge regarding the PA of children with asthma are mandatory. One of the possible responses to parental anxiety and the associated behavior could be to increase asthma knowledge, since a recent study suggested poor knowledge of asthma in parents, also emphasizing that increasing asthma knowledge among parents is advised [37].

Even though this study was comprehensive, a larger sample size may enable a more detailed approach to parental and child anxiety while considering various drug approaches in the treatment of asthma in children. Possible biases, including the personality traits of both children and their parents, current life circumstances in the family such as socioeconomic status and overall health-related behaviors, were not taken into consideration in this study. Since this research did not include the length of treatment since the first diagnosis, it is relevant to investigate its possible mediating role in the relationship between asthma severity and asthma-related anxiety. Future research should include an assessment of asthma control with the use of validated tools for pediatric patients, such as the Asthma Control Test (ACT) [38]. The advantage of this study was an in-person approach to all respondents and their children in an ambulatory setting while taking into account the severity of the asthma. The generalizability of the current findings could be significantly improved with a longitudinal methodological approach. 

## 5. Conclusions

The findings of this study show different experiences of asthma-related anxiety between children and their parents, as well as the influence of asthma severity on anxiety levels. Children most often experienced concerns related to physical activity (PA) and not being successful in sports or dancing due to asthma. Parents reported higher levels of anxiety regarding asthma attacks occurring without warning signs, the fear of side effects, the fear of their child missing out on school activities and the fear of asthma attacks during sports activities. This indicates the extreme importance of educating asthma patients and their parents on proper asthma treatment and on the relationships between physical activity and asthma and respiratory health. Healthcare providers should address the specific concerns of both children and their parents in asthma management, and interventions aimed at reducing asthma-related anxiety should take into account the individual needs and experiences of both the children and their parents.

## Figures and Tables

**Table 1:** Demographic data and clinical status of the patients (*n* = 150).

		Total Sample
Gender	Boy	78 (52%)
	Girl	72 (48%)
Age		10.9 ± 3.7
GINA stage	GINA stage 1	8 (5.3%)
	GINA stage 2	100 (66.7%)
	GINA stages 3 and 4	34 (22.7%)
	GINA stage 5	8 (5.3%)
Drug treatment	SABA if necessary, no permanent therapy	8 (5.3%)
	Montelukast	9 (6%)
	ICS	91 (60.7%)
	ICS and Montelukast	13 (8.7%)
	ICS and LABA	21 (14%)
	ICS and LABA and Montelukast	5 (3.3%)
	Biological therapy	3 (2%)
Asthma severity	Mild asthma	108 (72%)
	Moderate and severe asthma	42 (28%)
PAAS (*n* = 150)		1.51 ± 0.83
YAAS (*n* = 108)		1.02 ± 0.77

Continuous data are presented as arithmetic means ± SD; categorical data are presented as frequencies (percentage). Abbreviations: GINA, Global Initiative for Asthma; PAAS, Parent Asthma-Related Anxiety Scale; YAAS, Youth Asthma-Related Anxiety Scale.

**Table 2:** Comparison of the average values achieved on the PAAS and YAAS and subscales of the two examined groups of patients.

	Mild Asthma	Moderate and Severe Asthma	*p*
PAAS (*n* = 150)	1.44 ± 0.82	1.69 ± 0.83	0.092
Asthma severity and treatment subscale PAAS	1.46 ± 0.91	1.63 ± 0.94	0.314
Restrictions due to asthma symptoms subscale PAAS	1.4 ± 0.9	1.76 ± 0.9	0.032 ^a^
YAAS (*n* = 108)	0.92 ± 0.77	1.24 ± 0.75	0.047 ^a^
Asthma severity and treatment subscale YAAS	0.86 ± 0.77	1 ± 0.68	0.355
Restrictions due to asthma symptoms subscale YAAS	1.03 ± 1.04	1.72 ± 1.25	0.004 ^a^

^a^ Significantly different after comparing children and parents of children diagnosed with mild versus moderate and severe asthma, *t*-test for independent samples, homogeneous variances. Continuous data are presented as arithmetic means ± SD. Abbreviations: PAAS, Parent Asthma-Related Anxiety Scale; YAAS, Youth Asthma-Related Anxiety Scale.

**Table 3:** Differences in asthma-related anxiety among children and their parents in comparable items on the YAAS and PAAS (*n* = 108).

	Parent PAAS	Child YAAS	95% CI of the Difference		*p*
			Lower	Upper	
Fear of an asthma attack without warning	1.6 ± 1.3	0.9 ± 1	0.4	0.9	<0.001 ^a^
Fear of an asthma attack without medication	1.3 ± 1.3	1 ± 1.2	0	0.6	0.061
Fear of death	0.7 ± 1.1	0.4 ± 0.9	0	0.5	0.060
Fear of social anxiety due to an asthma attack	0.9 ± 1	1.1 ± 1.3	−0.5	0.1	0.108
Fear of side effects	1.6 ± 1.3	0.8 ± 1	0.5	1.1	<0.001 ^a^
Fear of missing out in school	1.5 ± 1.3	1.1 ± 1.4	0.1	0.7	0.006 ^a^
Fear of falling behind others	0.9 ± 1.1	1 ± 1.3	−0.3	0.3	0.904
Fear of an asthma attack during PA	2 ± 1.3	1.4 ± 1.4	0.3	0.9	<0.001 ^a^
Fear of impaired performance in sports	1.4 ± 1.4	1.3 ± 1.5	−0.2	0.4	0.524

^a^ Significantly different after comparing children and parents of children diagnosed with mild versus moderate and severe asthma, *t*-test for paired samples. Continuous data are presented as arithmetic means ± SD.

## Data Availability

The data presented in this study are available on request from the corresponding author. The data are not publicly available due to the privacy of the respondents.
